# Extramedullary Osseointegration—A Novel Design of Percutaneous Osseointegration Prosthesis for Amputees

**DOI:** 10.3389/fbioe.2022.811128

**Published:** 2022-02-10

**Authors:** Yingying Sun, Jinying Xu, Shuang Lv, Ziran Xu, Lisha Li, Yan Li, Yulin Li

**Affiliations:** ^1^ The Key Laboratory of Pathobiology, Ministry of Education, College of Basic Medical Sciences, Jilin University, Changchun, China; ^2^ Department of Stomatology, the First Hospital of Jilin University, Changchun, China; ^3^ Division of Orthopedics and Biotechnology, Department for Clinical Intervention and Technology (CLINTEC), Karolinska Institutet, Stockholm, Sweden

**Keywords:** amputation, implant, osseointegration, prosthesis, endoprosthesis, cortical bone, cancellous bone tissue, rehabilitation

## Abstract

The percutaneous osseointegrated (OI) prostheses have greatly improved the overall quality of life for amputees. However, the long-term maintenance of the OI prostheses is still challenging. A major problem is bone resorption around the bone-implant-skin interface, which might cause implant loosening or osteomyelitis. Another problem is the breakage of connecting components between the intramedullary implant and external prosthesis due to excessive stress. We designed a novel osseointegration implant by changing the bone-implant contact from the inner cortex to the outer surface of cortical bone. In the current study, we compared the extramedullary cap-shaped implants with the intramedullary screw-type implants in rabbits. Osteointegration was confirmed at the interface of bone to implant contact (BIC) in both implant types. The external implant induced intramedullary bone regeneration in the medullary canal and increased the cortical bone density at the end of the stump. This study provides a new perspective on the design of osseointegration implants which might prevent the currently reported complications of the intramedullary OI systems.

## Introduction

The clinical application of osseointegration (OI) prosthesis for amputees started in Sweden in the 1990s with screw-type implants (OPRA system) and emerged as a rapidly developing area in orthopedic surgery (Lenneras et al., 2017; Li and Brånemark, 2017). With OI surgery, a screw-shape or press-fit implant is inserted intramedullary into the bone stump (Pendegrass et al., 2006). The implant is then connected by transdermal components to the external prosthesis to provide a weight-bearing function (Holt et al., 2013). This technology avoids soft-tissue problems associated with traditional socket prosthesis and provides physiological weight bearing to skeleton. It also preserves a range of motion of the proximal joint under prosthetic usage and provides sensory feedback. All the above advantages contribute to the dramatic improvement in quality of life for amputees (Staubach and Grundei, 2001; Kang et al., 2010; Mastinu et al., 2019; Kim et al., 2020; Souza et al., 2020; Shevtsov et al., 2021).

The OI systems continually evolved during the past 30 years. A major breakthrough was the introduction of press-fit Endo-Exo- Prosthesis (EEPs) by Hans Grundei et al. who applied porous cobalt chromium metal stem as the intramedullary implant (Mittelmeier et al., 1997). Dr. Al Muderis and co-workers later developed the Osseointegrated Prosthetic Limb (OPL) system, in which the implant material was changed from cobalt-chromium to titanium (Thomson et al., 2019). The press-fit systems shortened the time for surgery and rehabilitation, becoming favorable choices for patients and surgeons in recent years (Hoellwarth et al., 2020).

Both screw-type and press-fit implants are inserted in the bone intermedullary canal. When the implant is performing a weight-bearing function, the surrounding bone cortex distal to the BIC will lack sufficient stress stimulation, resulting in bone resorption (osteolysis) around the bone-implant-skin interface (known as a stress-shielding effect) (Atsuta et al., 2016). The colonized bacteria from the skin opening might penetrate through the osteolytic bone and cause implant loosening and osteomyelitis (Chavrier and Couble, 1999; Rompen et al., 2006; Tillander et al., 2017). In the 5-year follow-up of the 51 patients with OPRA implants, 70 superinfections occurred in 34 patients (67%) and 14 deep infections occurred in 11 patients (22%) (Branemark et al., 2019). The results are similar to the 5-years retrospective study on 39 patients using the press-fit implants, in which thirty patients (77%) presented with 156 events of superficial infections in total, with eight events (5%) in 4 patients being classified as deep infections (Reetz et al., 2020).

Mechanical failures of the transdermal components are also of concern for the OI prostheses. In the OPRA cohort, the abutment and/or abutment screw fractures occurred in 15 of the 51 patients (29%) within 5 years (Branemark et al., 2019). Among the 39 patients using the ILP system, nine patients (23%) got 12 dual-adaptor breakages in 5 years (Reetz et al., 2020). A long-term follow-up of 111 patients using the OPRA system indicates that mechanical complications dramatically increased after 8 years of OPRA usage. There is also a positive relationship between a higher activity grade and the number of mechanical complications (Hagberg et al., 2020).

Unlike intraoral and craniofacial osseointegration, the functional structure of the limb stump provides the possibility of inducing osseointegration around the periosteum. Theoretically, the externally applied implant directly closed the bone canal. The implant itself is used as a connecting component to the external prosthesis, which potentially reduced the risk of connecting component breakage. In this study, the design of geometric shape of the external OI implants and the surgical approaches are described. The osseointegration at the bone-implant interface was analyzed and compared with the screw-type intermedullary systems. The process of differentiation of mesenchymal stem cells (MSCs) toward osteogenesis was also explored.

## Material and Methods

### Implant Design and Mechanical Processing

**FIGURE 1:**
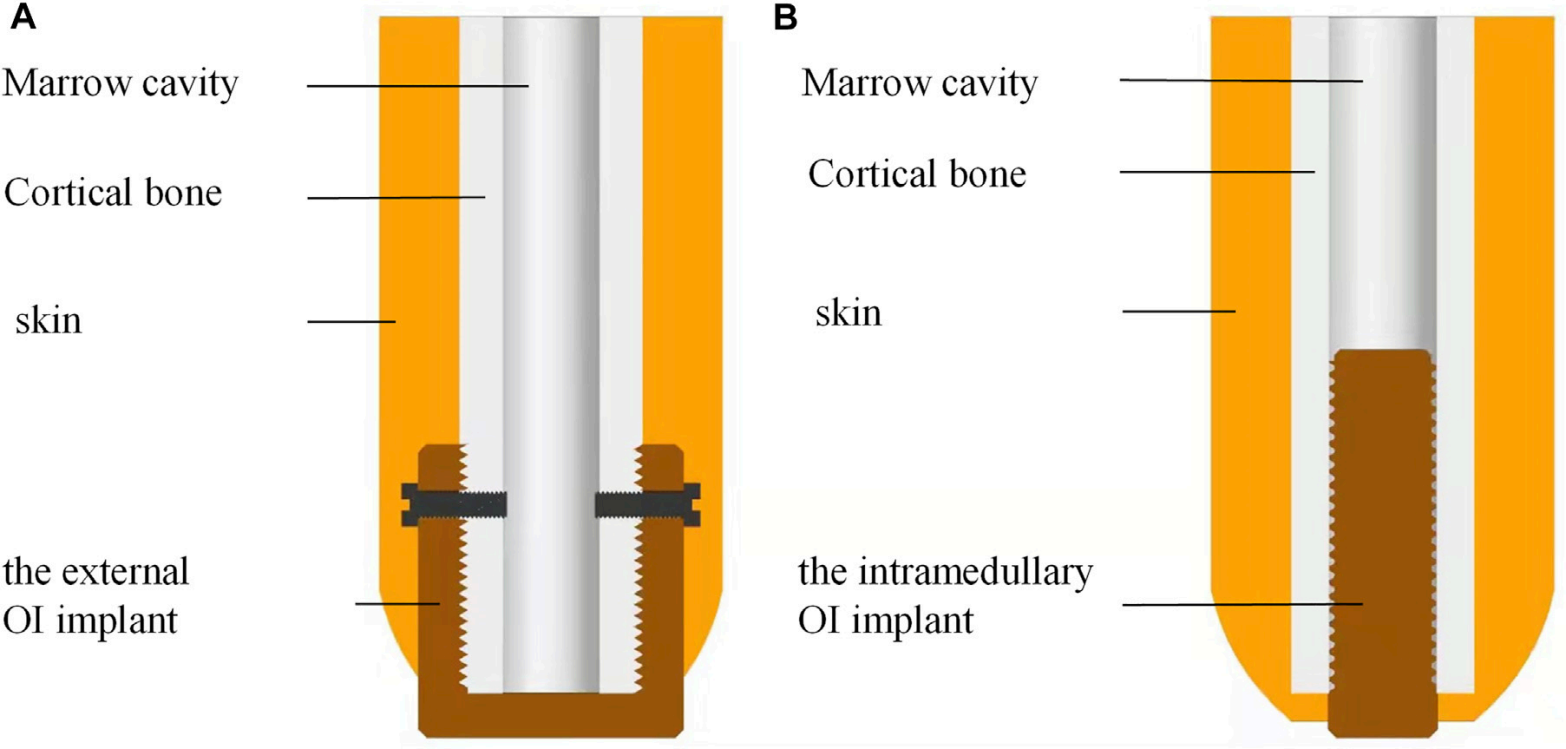
A schematic diagram of the novel external cap implant and the internal columnar implant. **(A)** Novel design of the extramedullary cap-shaped OI implant; **(B)** Current design of the intramedullary implanted columnar implant.

We designed the extramedullary cap-shaped OI implant (Figure 1A) and the intramedullary thread type implant, which mimic the OPRA systems as control (Figure 1B). Two types of implants were both 10 mm in length at the BIC with self-tapping screw threads.

**FIGURE 2:**
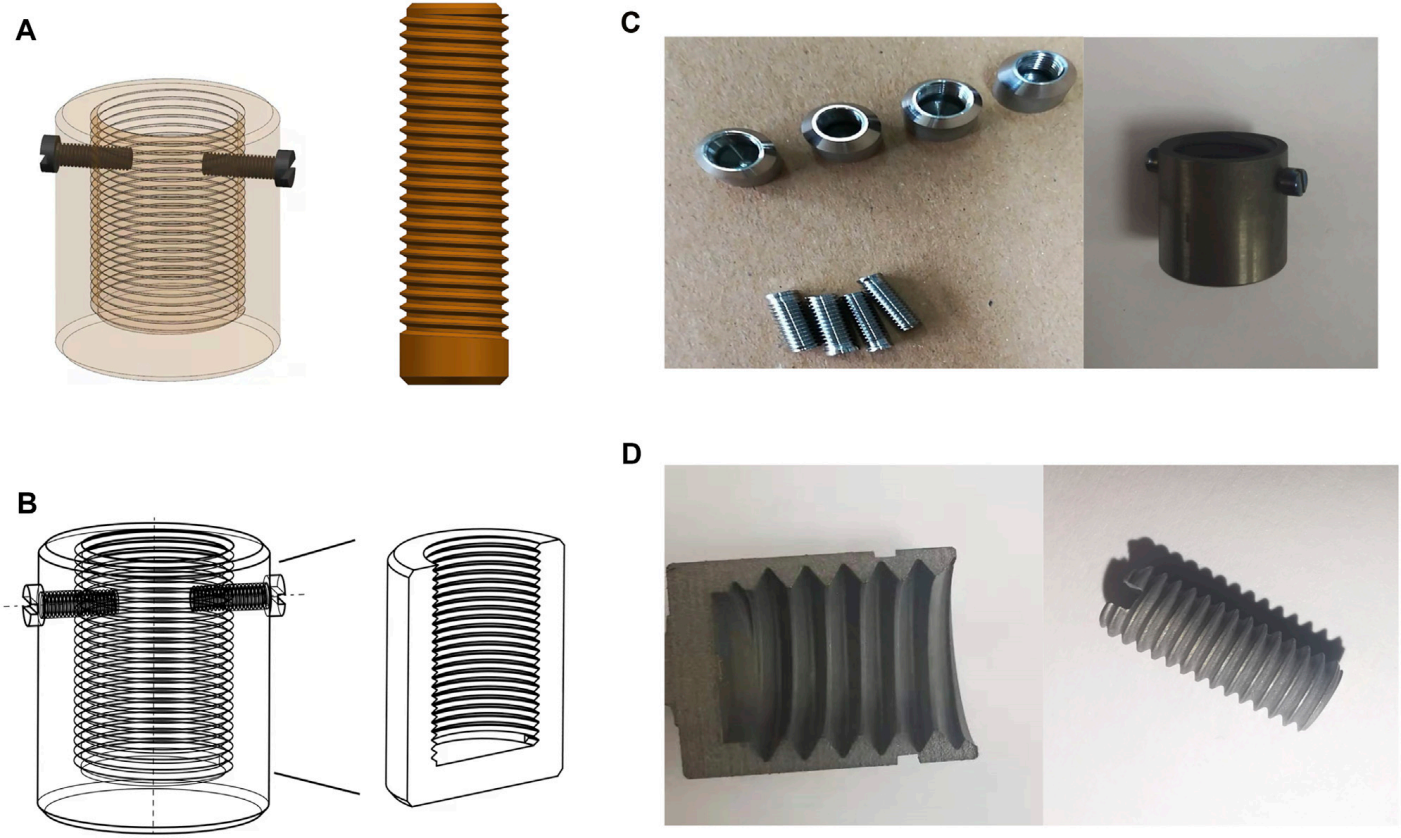
Three-dimensional model diagram and mechanical processing of implants. **(A)** 3D mechanical processing diagram of the external cap implant and the internal column implant; **(B)** the model diagram of the inner morphology of the external cap implant; **(C)** samples of mechanically processed external cap implant and internal columnar implant; **(D)** implant appearance with surface treatment by sandblasting acid etching.

The implant sizes were determined according to the geometric characters of the rabbit tibia. Figure 2A shows the 3D mechanical processing diagram, and Figure 2B shows the model diagram of the inner morphology of the external OI implant. According to the X-ray measurements of the rabbit tibia, the outer and inner diameters of the bone marrow cavities ranged from 7–8 mm and from 3–4 mm, respectively; therefore, the internal diameters of the external OI implant were set as three types: 7, 8, and 9 mm, and the outer diameters of the screw-type implant were 3, 4, and 5 mm. The screw threads of the surface in contact with the bone were designed with a standard M8 coarse pitch with a uniform length of 10 mm, and the bottom thickness of the external implant was 1 mm.

UG NX 10.0 software was used to draw the design draft in three dimensions. The implants were mechanically processed with the computer numerical control machine (JFMT360, Jinan, China). A titanium rod with the corresponding diameter and length was selected according to the designed parameters. The implant surface in contact with the bone was treated with sand blasting and acid etching. The sandblasting treatment was performed by using Al2O3 (110 μm) under 0.6 MPa at 10 mm distance and a 45° angle for 15 s. Hydrofluoric acid (40%) was applied for 60 s. After that, the implants were cleaned ultrasonically in anhydrous ethanol and deionized water for 20 min, dried, sealed in bags, and sterilized using high-temperature steam (120°C, 40 min).

### Three-Dimensional Finite Element Analysis of Implants

To evaluate the mechanical properties of the implants under loading, these two types of implants were analyzed under simulated stress *in vivo* using a three-dimensional (3D) finite element method. The 3D mechanical drawing software Pro/E Wildfire (Parametric Technology Corporation, Needham, MC, United States) was used to establish a 3D solid model of the titanium implant and generate a finite element mesh. Then, the software ANSYS Workbench17.0 (SAS IP, Inc., Cary, United States) was used to generate the finite element model and import the material parameters of titanium alloy (elastic modulus 110GPa, Poisson’s ratio 0.3) (Neumeister et al., 2017).

To simulate the force and deformation of the implant when the rabbit was walking under its body weight, a fixed support was applied around the bone. According to the body weight of the rabbit, 50 N perpendicular to the surface was applied to the bottom surface of the implants and 20 N was applied on the side of the implants. The parts subjected to the maximum stress were more likely to undergo deformation, but could not be damaged if the loading force does not exceed the maximum stress value. The material parameters were shown in Table 1.

**TABLE 1:** The material parameters in the three-dimensional (3D) finite element analysis.

Material parameter	Density	Poisson’s ratio	E-Modulus
Cortical bone	1228 kg/m^3^	0.3	1.7 E + 10 Pa
Implants	4620 kg/m^3^	0.36	9.6 E + 10 Pa

### Surgical Procedures With New Zealand Rabbits

Twenty tibial amputation models of ten New Zealand white rabbits (6 months, 2, 5 kg) were established and divided into two groups, implanted either type of the implants, with 10 samples in each group. Adequate procedures were conducted to minimize pain and discomfort in the animals. All animal trials were approved by the Ethics Committee of Experimental Animal Welfare of Jilin University [license number SCXK (Ji)-2016-0004] and were carried out in accordance with the regulations.

During surgery, sodium pentobarbital (3%) was slowly injected into the ear vein to anesthetize the rabbits. After shaving and cleaning (chlorhexidine 0.5 mg/ml) the recipient’s leg, the distal aspect of the tibia was exposed through a skin incision. The blood vessels were ligated and cut first, the muscle tissue and nerve were then annularly dissected to expose the central portion of the tibia where osteotomy was done with bone saw.

**FIGURE 3:**
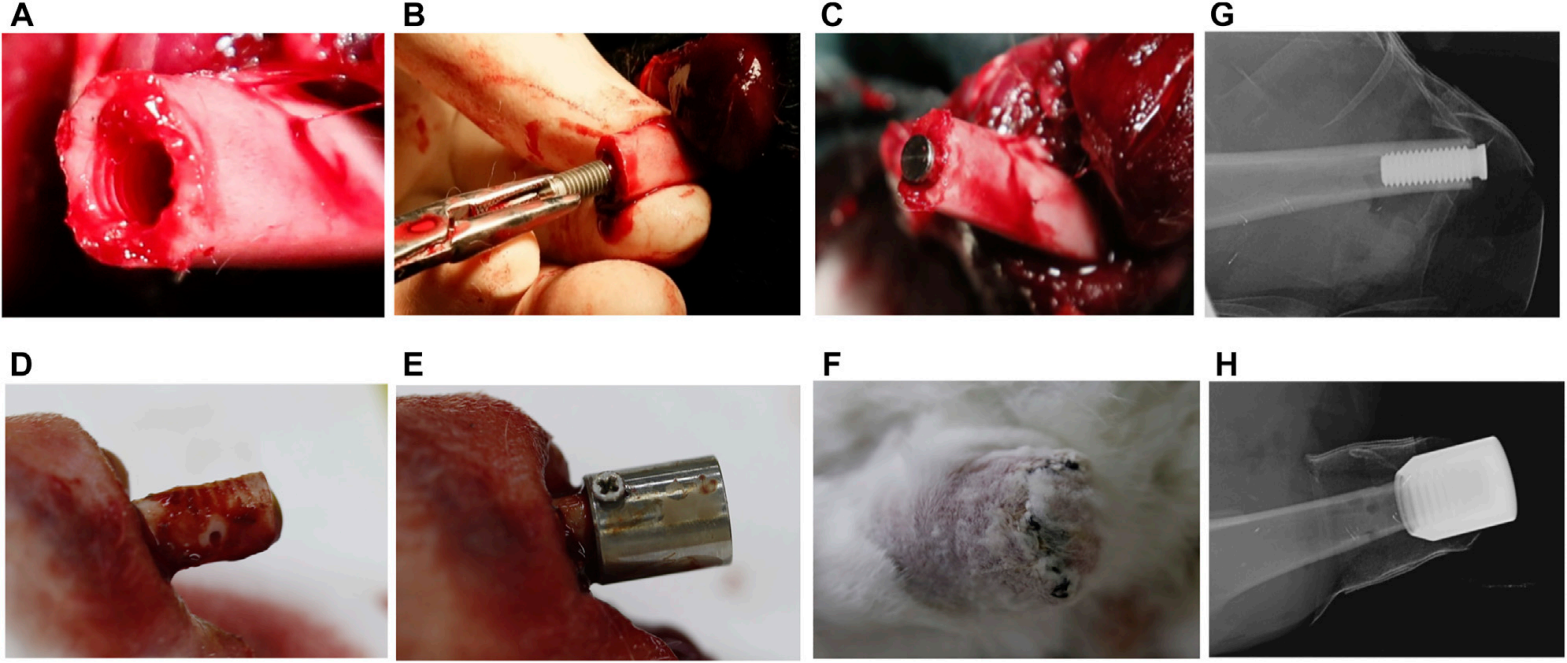
The surgical procedure of implantation in New Zealand Rabbits. **(A)** Preparation of the transplant bed before implantation of internal columnar implants; **(B)** the internal columnar implant was successfully implanted; **(C)** the columnar implant inside the medullary cavity; **(D)** preparation of transplant bed before implantation of external cap implants, with drilling holes on the cortical bone; **(E)** the external cap implants outside the medullary cavity; **(F)** the external cap implant was successfully implanted; **(G,H)** X-ray examination after surgery.

For the control group, the thread tap with a length of 10 mm was used to prepare the internal side of cortical bone (Figure 3A). The screw-type implant was then threaded into the bone canal until stability was achieved (Figures 1B, 3B).

For the external OI group, the periosteum and soft tissue were removed from the bone stump of 10 mm from the end of the amputation, and the screw threads were created on the outer cortical bone surface by thread tap (Figure 1A). Three to four holes were randomly drilled in the cortical bone to stimulate blood supply (Figure 3D). Titanium screws can be added on both sides of the proximal part of the implant to increase initial stability (Figure 3E). The periosteum was reclosed by suture as far as possible.

For both groups, the muscle endings were attached to periosteum and the implant/bone endings were buried under the skin (Figure 3F). Before the animals recovered from anesthesia, the implant sites of the stump were photographed using oral X-ray equipment (Figures 3G,H). Intramuscular injection of penicillin sodium was administered daily for 3 days, and the implants remained *in situ* for 3 weeks. Wound sites were checked daily in case of inflammation, infection, and implant loosening. All the wounds healed well without signs of infection.

### Micro-CT Scan

Twenty-one days after the operation, the experimental animals were euthanized with an excess of 3% sodium pentobarbital. The implants and tibial bone blocks with a length of approximately 20 mm were removed and placed in 4% paraformaldehyde solution for fixation. The specimen was fixed on the micro-CT fixing plate, the orientation was adjusted, and the specimen was placed in a micro-CT machine for scanning. The parameters were a scanning voltage of 80 kV, a current of 50 μA, and a layer thickness of 40 μm. The area of interest was set to a 0.5 mm circumferential volume range around the bone-implant interface. The original data were copied on micro-CT, and the image was processed using Mimics software. The bone mass at the bone-implant interface was calculated according to the CT value of 300–600.

### Histological Analyses

To examine new bone formation and osseointegration around the implants, bone blocks with implants were retrieved, and the surrounding bone was processed for histological analyses. The retrieved bone samples were placed in 70% ethanol for fixation and dehydration. Subsequently, the samples were infiltrated with 2-hydroxyethyl methacrylate light-curing resin (Technovit 7200 VLC; Heraeus Kulzer, Wehrheim, Germany), and were finally embedded in the same resin. Non-decalcified sections were then cut with an Exakt saw (Exakt Apparatebau, Norderstedt, Germany) parallel to the long axis of the implant and ground down to a thickness between 30 and 10 μm following the cutting-grinding technique. The sections were stained with toluidine blue–pyronin and hematoxylin-eosin (HE) dye, and then observed under a light microscope (Eclipse ME600; Nikon, Tokyo, Japan).

Immunohistochemical staining was performed using an anti-AP antibody (ab108337, Abcam) and an anti-Runx2 antibody (ab23981, Abcam). To expose the epitopes, the specimens were treated thrice with xylene, 2-Methosyethyl acetate, and acetone. To remove the superficial layers of methylmethacrylate, the specimens were rinsed with distilled water and incubated with TE or TR buffer (Dako). Before immunostaining, endogenous peroxidase and host immunoglobulin G (IgG) were blocked using peroxidase blocking reagent (Dako) and rodent block R (Biocare). The blocking reagents were removed, and primary antibodies were applied. Specimens were incubated overnight and rinsed with TTBS buffer and antibody detection rabbit-on-rodent HRP-Polymer (Biocare) for AP detection, as well as secondary anti-rabbit IgG (Dako) for Runx2 detection. After 60 min of incubation, immunohistochemical detection was performed using DAB Chromogen Systems (Dako). The samples were rinsed, counterstained with HE, and stored after dehydration. Isotype controls were performed with rabbit-on-rodent HRP-polymer or secondary anti-rabbit IgG only.

## Results

### The Implants and Three-Dimensional Finite Element Analysis

The implants were processed in accordance with the 3D structure drawn using UG NX 10.0 (Figures 2A,B). They had smooth surface after mechanical processing and appeared to be dark and coarse after classic sandblasting and acid etching (Figures 2C,D). After ultrasonic washing, the implants were dried and sterilized in a sealed bag.

**FIGURE 4:**
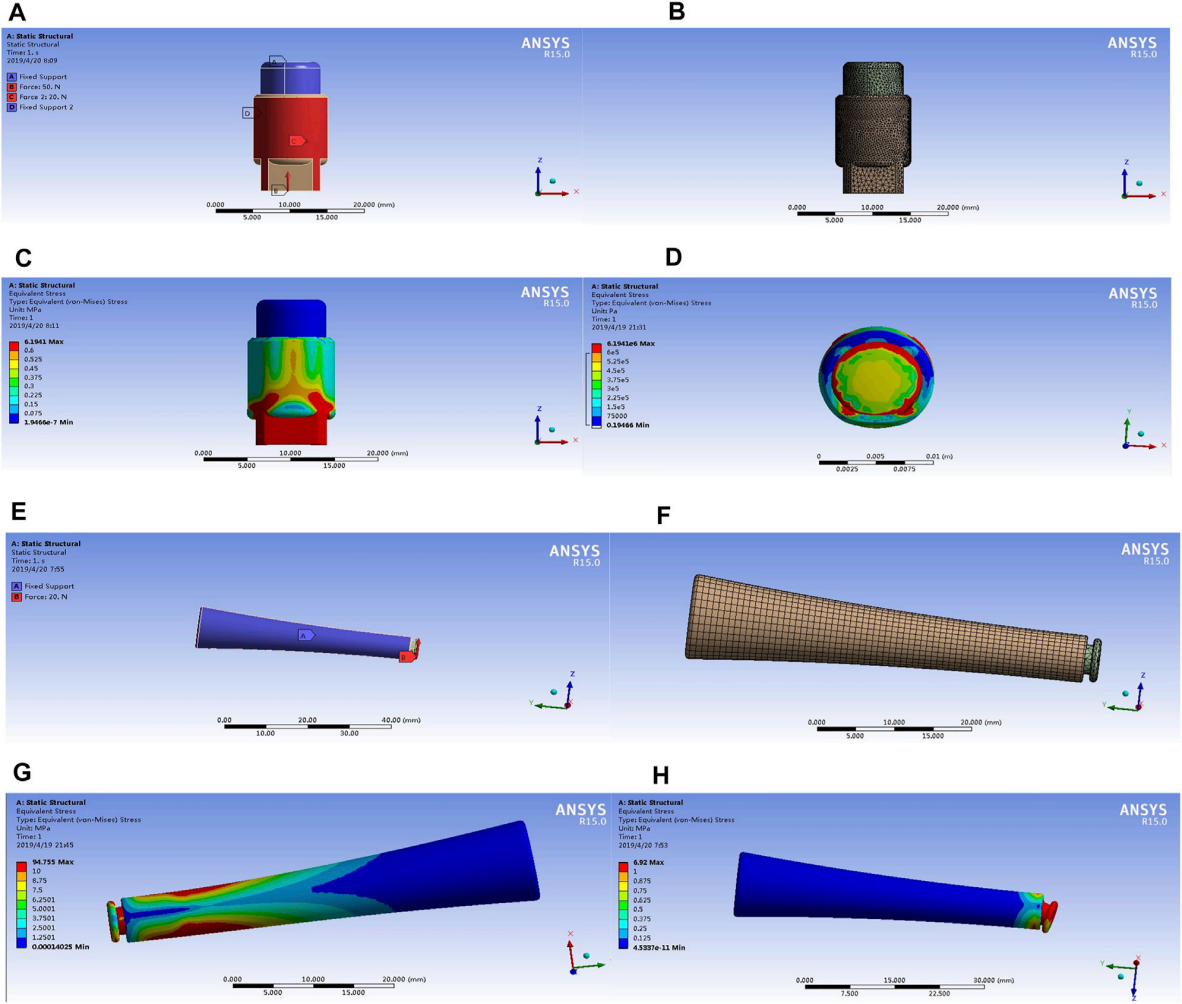
The stress analysis results of the implants with the three-dimensional finite element analysis. The results of the external cap implant were shown in Figures 3A–D; the results of the internal cap implant were shown in Figures 3E–H. Different colors in the color scale represent different stress values. The blue area represents the part with the minimum stress value, and the red area represents the part with the maximum stress.

The stress analysis results of the implants were shown in Figure 4, the color indicator scale on the left of the figure indicated that the tested part suffered an increasingly load stress, the red area indicated the maximum stress. The implant was prone to deform if a load exceeds this stress. For the external OI implant, the maximum stress concentration appeared at the implant-bone junction, which functioned as a mechanical transmission part and located at the skin opening (Figures 4A–D). For the screw-type implant (Figures 4E–H), the maximum stress concentration lies proximal to the intramedullary implant in contact with the medial surface of the cortical bone, and the connecting rod where the implant was connected to the external prosthesis, as shown in Figure 4G. In addition, the connecting rod of the implant was prone to deformation and fracture under the action of high stress concentration (shown as Figure 4H). This result is in accordance with the clinical reports that mechanical failures mostly occurred at the connecting components to the external prosthesis (Li and Fellander-Tsai, 2021). It is also in accordance with the proximal buttressing and distal bone resorption signs as often shown by X-ray analysis for patients who are using the bone-anchored prostheses (Tsikandylakis et al., 2014).

### Micro-CT Scan and Description After Implantation

**FIGURE 5:**
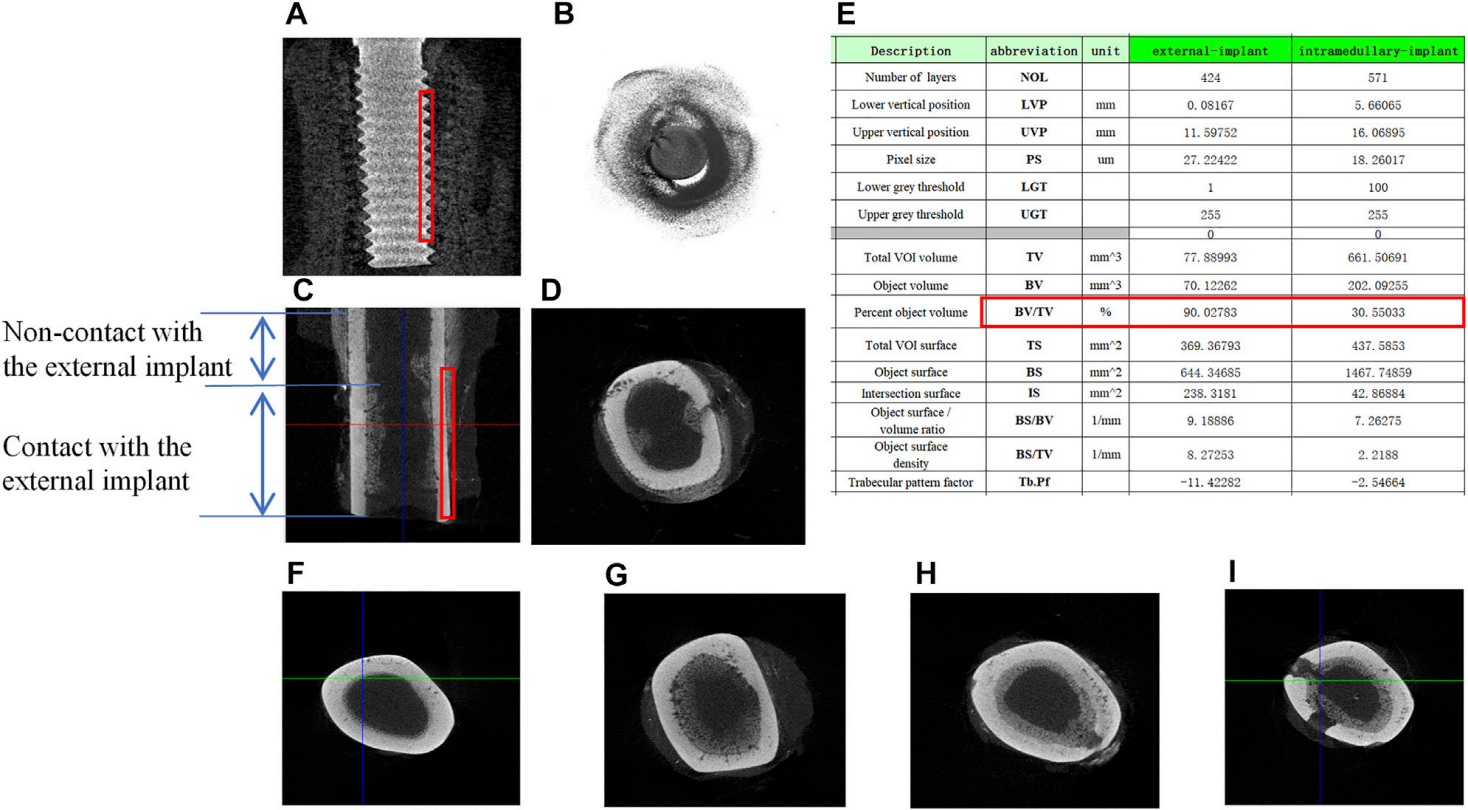
Micro-CT scan and the description of bone tissue after implantation. **(A)** Longitudinal scan of samples around internal columnar implants, the red boxes indicate the total VOI volume; **(B)** transverse scan of samples around internal columnar implants; **(C)** longitudinal scan of samples around external cap implants; **(D)** transverse scan of samples around external cap implants; **(E)** the description of Micro-CT scan results; **(F)** transverse scan of the normal rabbit tibia; **(G–I)** new bone regeneration in the marrow cavity surrounded by the external implant.

Micro-CT scan was performed to observe osseointegration (OI) at the implant-bone interface. For the screw-type implant, a close contact with the internal bone cortex was seen by both longitudinal and transverse tomography (Figures 5A,B). For the external OI implant, besides bone formation close to the external bone cortex, the new bone was also formed in the end of bone marrow cavity (Figures 5C,D). In addition, proximal to the BIC surface, sub-periosteum osteogenesis occurred onto the external surface of the cortical bone, but there was no new bone formation inside the marrow cavity (Figures 5C,D). From a transverse sectional view of the bone, Figure 5F showed the normal tibial canal without any bone hyperplasia. Figures 5G–I showed a significant new bone formation at the BIC outside the cortical bone, or inside the marrow cavity, including the pores prepared on the cortical bone.

The red box in Figures 5A,C showed the volume-of-interest (VOI) of the samples in 3D volume of the 0.5 mm radius ring around the implants. Micro-CT software was used to analyze bone regeneration in the VOI, and the average data were shown in Figure 5E. Among the important description obtained, the relative bone volume fraction (BV/TV) reflects the relative bone volume in the region of interest, which was positively correlated with the volume of the new bone. In the VOI of the bone with intramedullary implant, the value of BV/TV obtained was 90.02%, while that of the bone with extramedullary implant was 30.55%, indicating that the volume of the new bone at the BIC was less than that of the traditional intramedullary implants.

### Histological Analysis of Bone Tissue Around Implants

#### HE Staining of Undecalcified Bone Tissue Around the Implants

**FIGURE 6:**
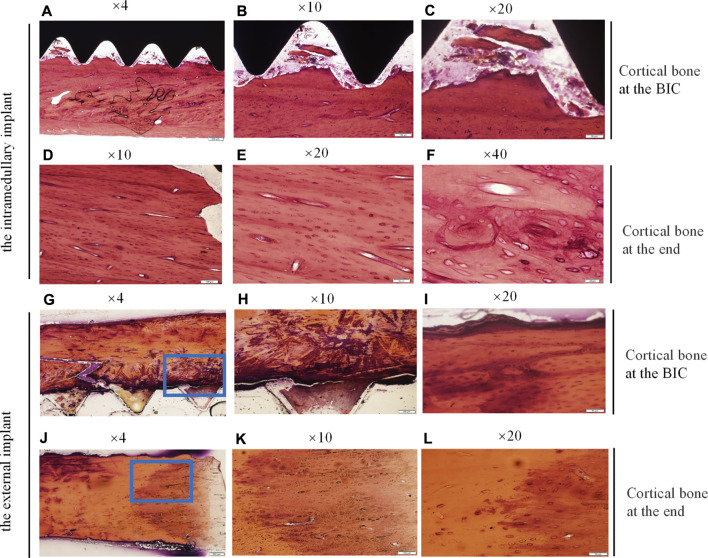
Histological sections and HE staining of the undecalcified bone samples around the implants, observed using a histological microscope. (magnification ×10, ×20, ×40). **(A–C,G–I)** New bone formation within the threads of both implants at the BIC; **(D–F,J–L)** the cortical bone at the end of the stump. Osteocyte lacunae was visible.

Bone samples were obtained 21 days after the intramedullary implant and the external cap implant grafted, undecalcified tissue sections and HE staining were performed on both samples around the implants. The results were shown in Figure 6. Histological images gradually magnified were shown from left to right, and titanium implants were shown in black or removed. Figures 6A–C and Figures 6G–I represent the observation areas at the BIC interface. Figures 6D–F and Figures 6J–L showed the cortical bone at the end of the bone stump.

Figures 6A–C showed the interface between the implant and bone, depicting the newly mineralized bone matrix in the threads, indicating successful osseointegration at the BIC interface. Figures 6G–I showed the interface between the inner thread surface of the external implant and bone, indicating that the new bone matrix in the thread was not obvious, which may not provide the best binding interface in the section. Figures 6G–I showed the increase of osteocytes in cortical bone and the appearance of vascular structures under high magnification. Figures 6D–F and Figures 6J–L showed the cortical bone tissue at the end of the stump. The area in contact with the bottom of the external implant was shown in Figures 6J–L. There were a large mass of bone cells and new bone trabeculae in this area, and the bone density was significantly increased. This increase in bone density was consistent with the direction of stress distribution at the implant. These results indicated that there was lower osseointegration at the BIC interface of the external implant as compared with the internal implant. However, the external implant might promote hyperplasia of proximal bone tissue without distal bone resorption.

#### Toluidine Blue Staining of Undecalcified Bone Tissue Around the Implants

**FIGURE 7:**
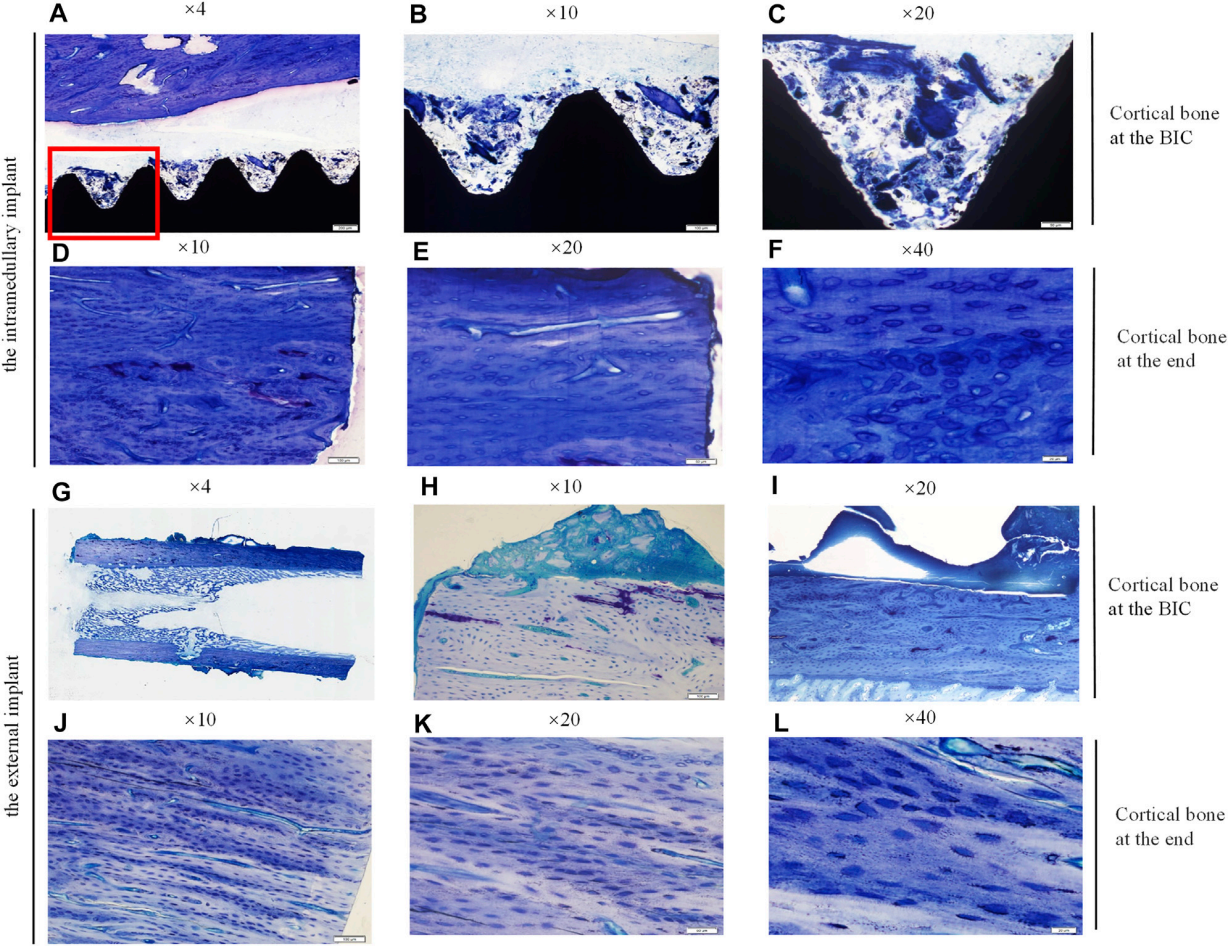
Histological sections and toruidine blue staining of the undecalcified bone samples around the implants, observed using a Histological microscope (magnification ×4, ×10, ×20, ×40). **(A–C,G–I)** New bone formation within the threads of both implants at the BIC; **(D–F,J–L)** the cortical bone with more dense osteocytes at the end of the stump. Osteocyte lacunae was visible.

Similarly, the bone samples around the implants were treated with undecalcified ground sections, and then stained with toluidine blue. The results were shown in Figure 7. Histological images at ×4, ×10, and ×20 magnification were shown from left to right, and the honeycomb tissue with blue staining was mineralized bone matrix. The results of the toluidine blue staining were consistent with those of HE staining. The results showed that cortical bone density increased, and bone regeneration occurred in the bone marrow cavity of the residual limb corresponding to the external implant. However, bone integration rates at the BIC interface were lower.

**FIGURE 8:**
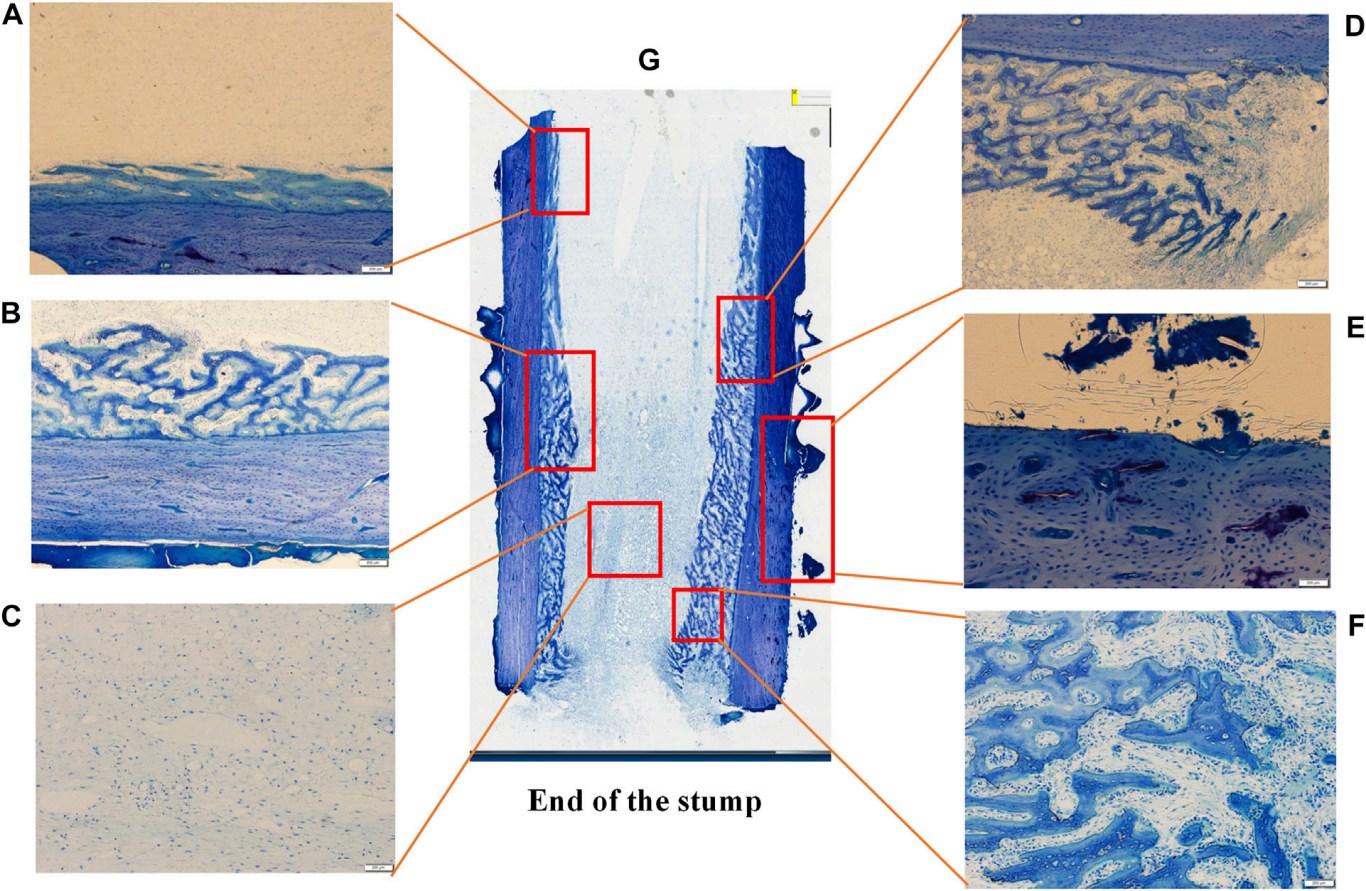
Histological sections and toruidine blue staining of the undecalcified bone sample after the external cap implant removed, observed using a Histological microscope (magnification ×4). Images **(A F)** were the magnification of the areas in the red box in image **(G)**, showed the bone formation in different areas.

Undecalcified sections and toluidine blue staining were performed on the bone blocks after implant removing (Figure 8). The blue-stained honeycomb tissue was the mineralized bone matrix. Figure 8G showed the overall histological morphology of the bone surrounded by the external implant. A large amount of bone regeneration was observed in the marrow cavity which externally attached to the implant. However, there was no osteogenesis in the bone marrow cavity far away from the implant, but there was considerable bone overgrowth outside the cortical bone.

Figures 8A–F showed the histological morphology of different regions at high magnification, showing the formation of new bone within the marrow cavity. The histological morphology was consistent with the results of the micro-CT.

#### HE Staining of the Decalcified Paraffin Sections

**FIGURE 9:**
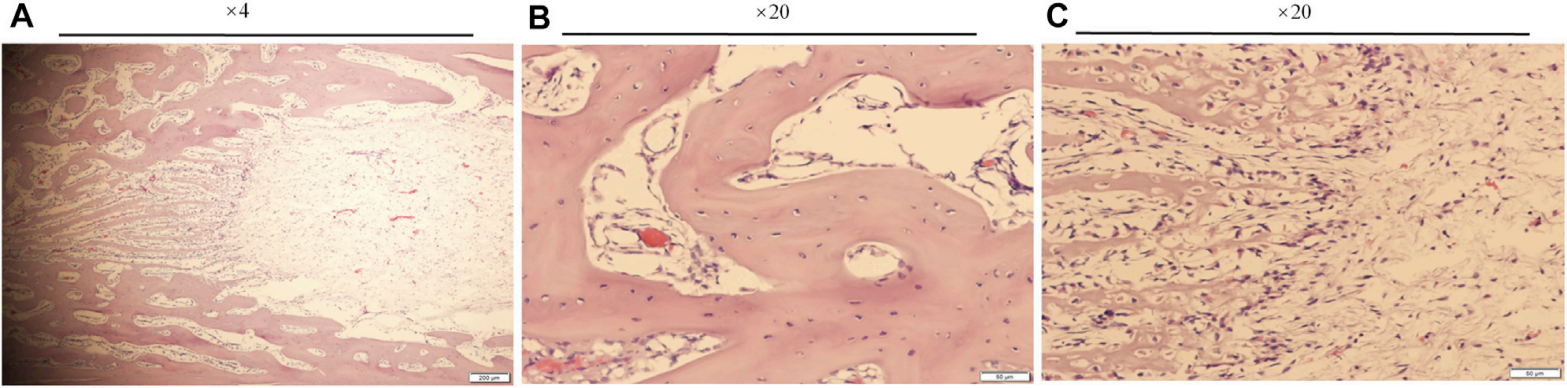
The paraffin sections and HE staining of the bone sample 21 days after the external cap implant grafted, observed using a Histological microscope (magnification ×4, ×20). These images from **(A–C)** were different areas of a sample from the distal to the proximal, at different magnifications gradually.

HE staining of the decalcified paraffin sections showed obvious bone neogenesis with mineralized bone matrix, bone trabeculae, and osteocytes around the endosteum of bone enveloped by the external implant (Figure 9). These images from A to B, to C are different regions of a sample from the distal to the proximal, at different magnifications gradually. Bone regeneration in the bone marrow cavity near the end of the stump was obvious, and the bone cortex proximal to the bone-implant junction was significantly denser (Figure 9A). At high magnification (Figure 9B), a mass of honeycomb bone trabeculae can be clearly seen in the marrow cavity. Regions in the bone marrow cavity gradually away from the end of the stump showed less significant new bone regeneration (Figure 9C).

#### Immunohistochemical Staining of the Decalcified Paraffin Sections

**FIGURE 10:**
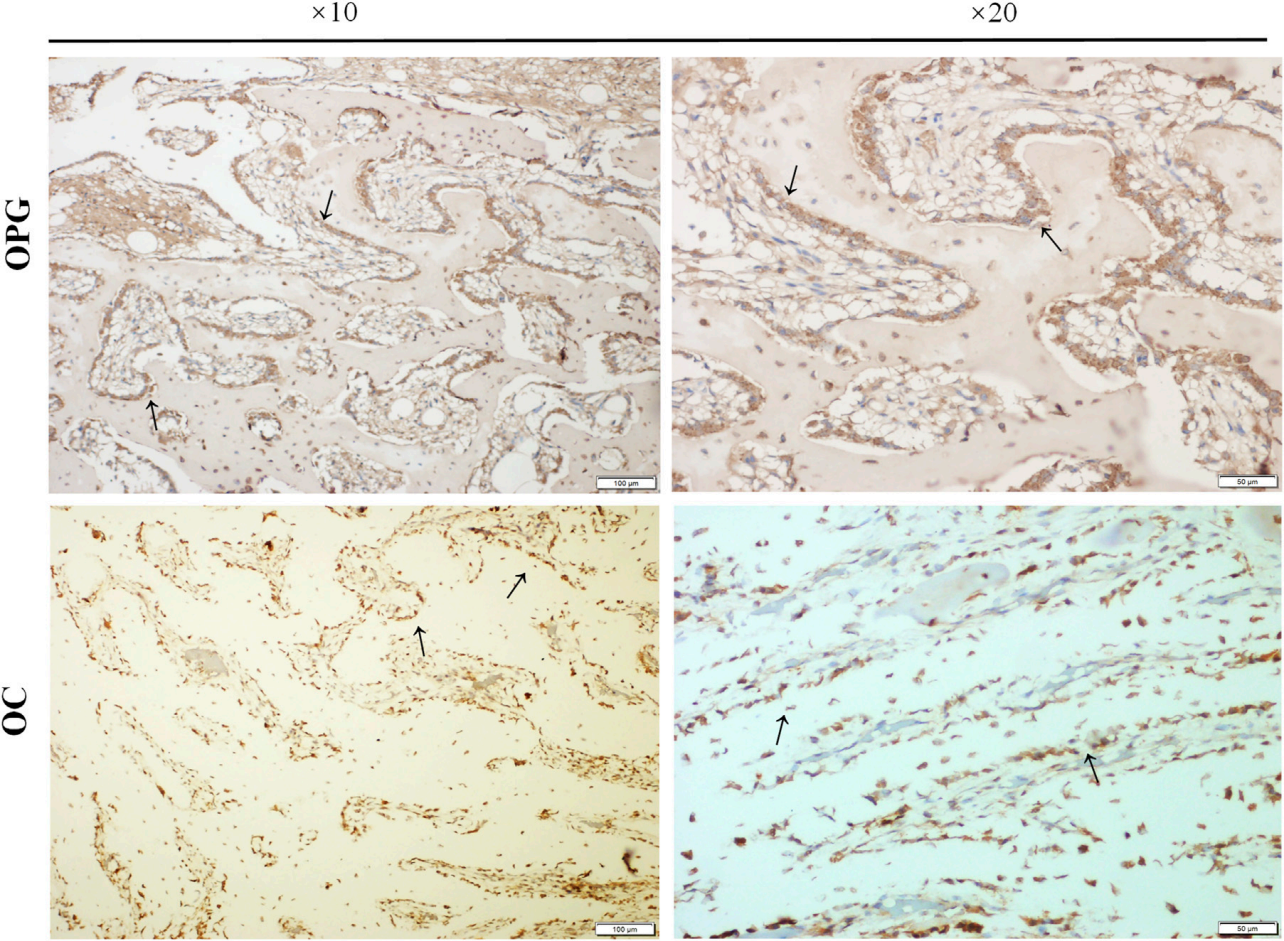
The expressions of osteogenic markers. Paraffin sections of bone samples 21 days after the external cap implant grafted, and the expressions of osteogenic markers including Osteoprotegerin (OPG) and Osteocalcin (OC) were detected by immunohistochemical staining. The black arrows indicated positive expression in the columnar osteoblasts arranged in a palisade.

To further confirm the osteogenic differentiation around the external implants, the expression of osteogenic markers, including osteoprotegerin (OPG) and osteocalcin (OC), was detected by immunohistochemical staining. The results were shown in Figure 10. The black arrow represented positive expression of OPG and OC, new bone was observed in the marrow cavity surrounded by the implant, and columnar arrangement osteoblasts were found. .

**FIGURE 11:**
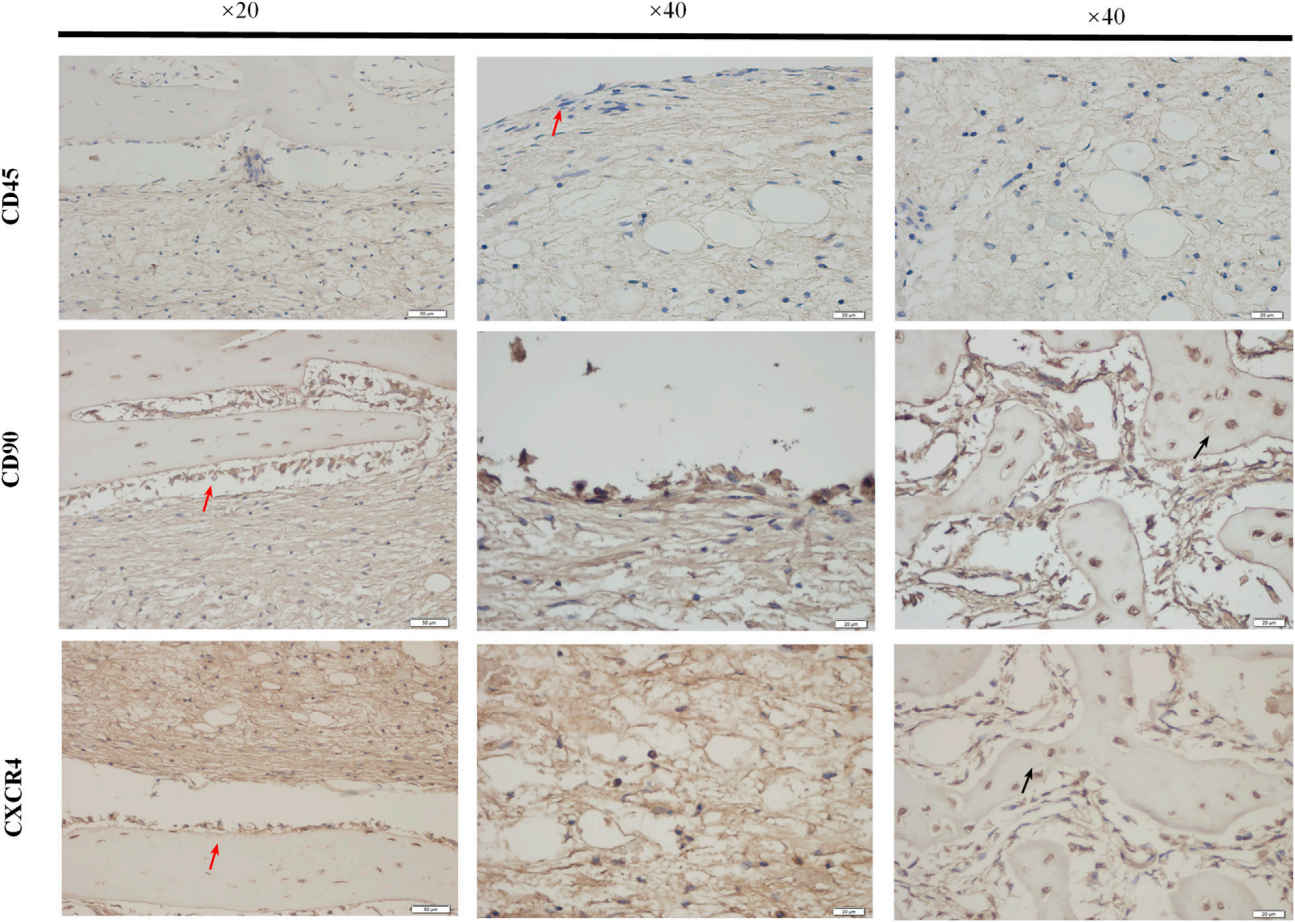
The expressions of BMSCs markers. The paraffin sections of bone samples 21 days after the external cap implant grafted, and the expressions of BMSCs markers (CD45, CD90) chemokines CXCR4 were detected by immunohistochemical staining. The brown-yellow particles indicated the positive expression. The expression of CD45 was negative and that of CD90 was positive, while the expressions of BMSCs chemokines CXCR4 were also positively expressed.

Immunohistochemical staining was used to detect the expression of Bone Marrow Stromal Cells (BMSCs) markers CD45 and CD90 and their chemokine CXCR4 in the bone tissue surrounded by the external implants, as shown in Figure 11. The red arrow indicated the endosteum in the medullary cavity at the implant contact area. The expression of CD45 was negative, CD90 was positive, and CXCR4 was positively expressed. The black arrows indicated positive expression in bone stroma.

**FIGURE 12:**
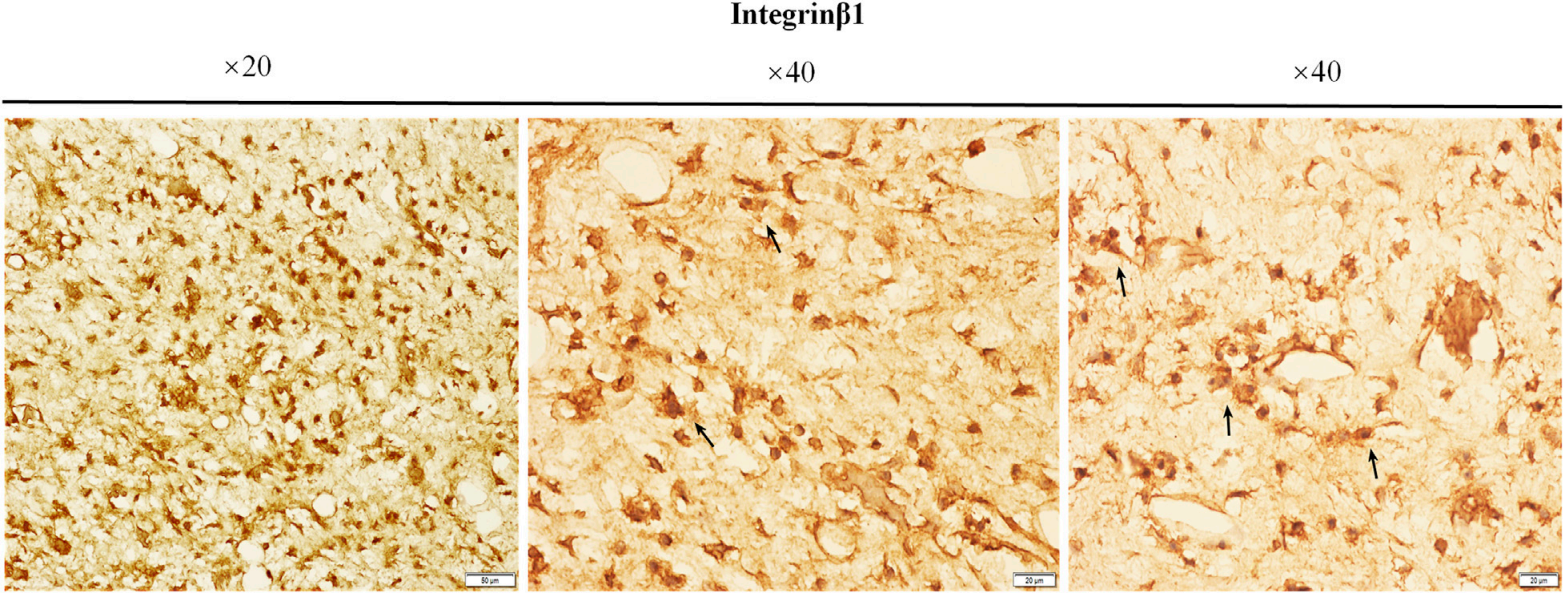
The expressions of integrin β1. The paraffin sections of bone samples 21 days after the external cap implants grafted, and the expression of integrin β1 was detected by immunohistochemical staining. The brown-yellow particles indicated the positive expression of integrin β1.

Immunohistochemical staining was used to detect the expression of integrin β1 in the bone tissue surrounded by the external implants, as shown in Figure 12. The brown-yellow particles indicated positive expression of integrin β1.

## Discussion

### Design Concept of the External OI Implant and Surgical Method

The long-term maintenance of the bone-anchored prostheses is challenging due to the risk of mechanical failures and deep infections (Li and Fellander-Tsai 2021). Previous efforts were mainly focused on improving the osseointegration efficiency by the modification of implant surface morphology, changing of implant materials, or improving surgical methods (Overmann and Forsberg 2020). Our 3D finite element analysis revealed that the two maximal stress areas in the screw-type implant system located proximally to the BIC and the connecting rod to external prostheses. Such a biomechanical profile well explained the frequently reported complications such as distal bone resorption around the bone-implant-skin opening and the deformity/fractures at the connecting components for the currently used OI systems (Figures 4E–H).

Unlike the intraoral and craniofacial osseointegration, the structure characters of the residual limb make the application of OI implant surrounding the bone ending possible. Our 3D finite element analysis indicated that the external application of implant not only closed the bone canal but also changed the stress-loading character of the system. The maximal stress was around the proximal bone-implant junction where the skin opening located (Figure 4C). We suppose that the local stress might induce bone hypertrophy at the implant-bone-skin interface, in favor of bacteria prevention under long-term prosthetic usage. However, it is unclear whether this stress pattern might also increase fracture risk around the bone-implant junction.

As shown in our study, the diameters of the external implants were significantly larger than the internal implants (7, 8, and 9 mm for the external implants and 3, 4, and 5 mm for the internal implants). With the same implant length, the external implant had therefore larger BIC surface for osseointegration and might contribute to implant stability. The external OI implant can also be directly connected to the external prostheses, which avoid the usage of connecting components and the related complications.

Single-stage surgery reduced the operating time, intraoperative bleeding, hospital stay, and overall costs for the osseointegration treatment. Application of single-stage surgery using intramedullary implant is controversial due to concern of bacteria penetration through the bone-implant-skin opening into the medullar cavity. The external OI implant induces osseointegration on the external part of bone cortex and thus avoids the bacteria penetration in bone cavity, reducing the concern for single-stage surgery.

### Osseointegration at the BIC and Bone Remodeling in the Medullary Cavity

Osseointegration is a dynamic status maintained by bone remodeling. The periosteum and endosteum are both layers of connective tissue on the surface of bone matrix, which is composed of osteoblasts and undifferentiated cells. They both have strong ability for bone regeneration and participate in bone remodeling. The blood supply to the surgical site is another prerequisite for Osseointegration and bone regeneration. Neovascularization can maintain the surrounding cells with the necessary oxygen and nutrients to promote their proliferation and differentiation (Abshagen et al., 2009). The arterial blood supply of long bones comes from three main sources: the nourishing arteries branch off to the periosteum, and the epiphyseal and metaphyseal arteries supply both ends and anastomose to the branch of the trophoblast arteries. However, the vessels in the periosteum are transverse, supplying the superficial layers of the cortical bone and usually do not penetrate into the deep layers (Strachan et al., 1990). The existing OI implants are fixed in the intramedullary canal, which occupies in the bone canal and damages bone marrow tissue and endosteum while blood supply to the periosteum is preserved (Greksa et al., 2012). In external implantation, the periosteum is stripped and direct blood supply to the BIC surface from the periosteum is blocked by the implant. Whether osseointegration can occur in this incapsulated environment is largely unclear.

The micro-CT images showed new bone formation at the BIC interface of both implant types. However, the VOI (0.5 mm around the implant) analysis showed that the BIC interface of the intramedullary implant is associated with larger amount of newly formed bone tissue and higher relative bone volume fraction BV/TV (A common index for the evaluation of bone mass of cortical bone and cancellous bone, often used for the evaluation of the bone interface bonding of the implant) (Meakin et al., 2017). This result indicates that the extramedullary osseointegration, although indeed occurred, was less efficient as the intramedullary osseointegration in our experiments. The reason might lie in the over-stripping of periosteum during operating procedures, which devoid large amount of bone progenitors or the blockage of blood supply from the periosteum. However, we found that for areas where direct bone-implant contact occurred, the formation of new bone at the BIC was as good as the internal implants. This result indicates that the blood supply from the penetrating vessels from the marrow cavity or bore holes might be sufficient for implant osseointegration. The major reason for the low BV/TA volume fraction lied probably in the geometric feature of the rabbit tibia. As in humans, the shaft of tibia in a rabbit is an oblate or triangle form. The contact between the implant and the outer surface of the cortical bone was not complete and significant gaps remained (Figure 5D). We suppose that the application of the external OI implant to femur, which is relatively round at the outer surface, might increase the BV/TV volume fraction. Further studies in larger animals are therefore needed for femur external osseointegration.

A favorable finding in this study is the new bone formation in the distal part of the intermedullary canal, which is encapsulated by the external implants (Figure 5C). This is a unique feature for the external implant system and we suppose that the intramedullary bone regeneration might increase the mechanical strength of the implant system during longer term of prosthetic usage.

### Titanium Stimulates Osteoblastic Differentiation of BMSCs to Achieve Osseointegration

To understand the cellular and molecular mechanism for intramedullary bone regeneration and extra cortical osseointegration around the external OI implant we performed a series of histological studies.

Consistent with the micro CT findings, histological analysis showed that the external OI implant not only avoided bone resorption at the implant-bone junction but can also promoted bone regeneration in the end of marrow cavity (Figures 6, 7). Osteoprotegerin (OPG) and osteocalcin (OC) were significantly positive, confirming an active bone regeneration process inside the medullary cavity (Figure 10). The mice and human-derived BMSCs expressed CD44, CD90, and CD105, and did not express CD45, CD34 (Koppula et al., 2010; Yan et al., 2019). In this study, CD90^+^ and CD45^−^ were selected as identification markers of BMMCs. To prove whether the implant recruits the BMSCs, we evaluated the expression of a major chemotactic receptor for BMSCs recruitment, the (C) C-X-C chemokine receptor type 4 (CXCR4) (Matsuura et al., 2010; Karazisis et al., 2017). As shown in Figure 11, BMSCs were recruited and differentiated into osteoblastic progenitor cells *in vivo*. The above immunohistochemistry results indicated that various factors, including chemokines secreted by osteoblasts adhering to the surface of the material, mobilized the surrounding mesenchymal cells and osteoprogenitor cells to participate in bone regeneration.

The areas with intramedullary bone regeneration had very limited contact with the external implant (only at the end or through the bore holes on cortex). Further studies were done to understand whether this type of distal osteogenesis was natural tissue reaction following amputation or it was induced by the titanium implant, which might provide spatial scaffold for the growth of blood vessels and the formation of new bone.

Integrin β1 is usually expressed in the cell membranes of bone marrow mesenchymal stem cells and osteoprogenitor cells (Du et al., 2011) and has been shown to be crucial in osteoblast adhesion (Gronthos et al., 1997) (Keung et al., 2011). Our results showed that the expression of integrin β1 was higher in the bone marrow cavity of the implant contact region, suggesting that integrin β1 plays a key role in active bone regeneration in this region (Figure 12). Contact with titanium surface probably induced osteoblastic differentiation of BMSCs by increased expression of integrin β1 the process of osseointegration and new bone formation in the medullary cavity.

In summary, our study for the first time evaluated the possibility for external osseointegration in animal amputation models. The results for new bone formation at the bone-implant-skin opening area and the distal bone canal were promising. However, the efficiency of the external osseointegration was not as satisfied as internal systems. Studies using larger animals with prosthetic bearing and long-term follow-ups are needed to further evaluate this novel concept for bone-anchored prosthesis.

## Data Availability

Datasets are available on request: The raw data supporting the conclusions of this article will be made available by the authors, without undue reservation. Requests to access the datasets should be directed to sunyingying@jlu.edu.cn.
